# Boards of Examiners

**Published:** 1900-07

**Authors:** 


### BOARDS OF EXAMINERS.

VIRGINIA STATE BOARD OF VETERINARY MEDICAL EXAMINERS. Drs. Harry Bannister, Roanoke; H. L. Drake, Leesburg; Wm. H. Bolyn, Lincoln, and Wm. T. Gilchrist, Norfolk, have recently been appointed on the Virginia State Board of Veterinary Medical Examiners, in the place of Drs. G. C. Faville, Norfolk; Charles McCulloch, Jr., Howardsville; Alex. J. Burkholder, Staunton, and E. P. Niles, Blacksburg. Dr. Thomas M. Sweeney, of Richmond, succeeds himself. The officers of the board are Dr. W. T. Gilchrist, president, and Dr. H. Bannister, secretary.

NORTH DAKOTA STATE BOARD OF VETERINARY MEDICAL EXAMINERS. The annual meeting of this board was held at Grand Forks, N. Dak., April 11, 1900. Meeting called to order in Hotel Dacotah, by the president, Dr. E. J. Davidson. Members present, Drs. Fisher, Grandin, and E. J. Davidson, Grand Forks. Dr. Sheppard, the secretary and treasurer, not being able to attend, the examinations were conducted by Drs. Davidson and Fisher. Two candidates, D. D. McNaughton, of Devil's Lake, and J. W. Hagerman, of Walwalla, passed a very satisfactory examination and were issued certificates to practice in the State. No further business, the meeting adjourned.

MARYLAND STATE BOARD OF VETERINARY MEDICAL EXAMINERS. At the annual meeting of this board, at Baltimore, Md., in June, the following veterinarians were examined and granted the license of the board: F. B. Berger, V.M.D., Baltimore; Glenn W. Horner, V.M.D., Finksburg; Lawrence A. Nolan, V.M.D., Baltimore, and Hulbert Young, V.M.D., Washington, D. C.

MICHIGAN STATE VETERINARY BOARD. This board held its first meeting for examination for applicants, February 6, 1900, at which time the secretary was recommended to issue certificates to thirty-five graduates and two non-graduates. The next meeting of this board will be held August 7, 1900.

IOWA STATE VETERINARY BOARD. In accordance with the Act recently passed by the State Legislature, the Governor has appointed the following well-known veterinarians on the Board: Drs. S. H. Johnson, Carroll; H. E. Talboth, Des Moines, and W. A. Heck, Maquoketa.

---

THE following appointments have been made as an Executive Committee of the Alumni Association of the American Veterinary College for the year 1900–1901: W. Horace Hoskins, '81, Chairman; Charles S. Atchison, '98; Harry E. Bates, '89; Charles E. Clayton, '93; Roscoe R. Bell, '87; William J. Coates, '77; Theodore A. Keller, '92.

